# Modulator-Dependent RBPs Changes Alternative Splicing Outcomes in Kidney Cancer

**DOI:** 10.3389/fgene.2020.00265

**Published:** 2020-03-26

**Authors:** Yang Wang, Steven X. Chen, Xi Rao, Yunlong Liu

**Affiliations:** ^1^Department of Medical and Molecular Genetics, Indiana University School of Medicine, Indianapolis, IN, United States; ^2^State Key Laboratory of Biocatalysts and Enzyme Engineering, School of Life Sciences, Hubei University, Wuhan, China

**Keywords:** alternative splicing, RNA-binding protein, modulation, cancer, dysregulation

## Abstract

Alternative splicing alterations can contribute to human disease. The ability of an RNA-binding protein to regulate alternative splicing outcomes can be modulated by a variety of genetic and epigenetic mechanisms. In this study, we use a computational framework to investigate the roles of certain genes, termed modulators, on changing RBPs’ effect on splicing regulation. A total of 1,040,254 modulator-mediated RBP-splicing interactions were identified, including 137 RBPs, 4,309 splicing events and 2,905 modulator candidates from TCGA-KIRC RNA sequencing data. Modulators function categories were defined according to the correlation changes between RBPs expression and their targets splicing outcomes. QKI, as one of the RBPs influencing the most splicing events, attracted our attention in this study: 2,014 changing triplets were identified, including 1,101 modulators and 187 splicing events. Pathway enrichment analysis showed that QKI splicing targets were enriched in tight junction pathway, endocytosis and MAPK signaling pathways, all of which are highly associated with cancer development and progression. This is the first instance of a comprehensive study on how alternative splicing outcomes changes are associated with different expression level of certain proteins, even though they were regulated by the same RBP. Our work may provide a novel view on understanding alternative splicing mechanisms in kidney cancer.

## Introduction

Renal cell carcinoma (RCC) is a common malignancy, representing 4.2% of all new cancer cases, with about 73,820 new cases and 14,770 deaths estimated for 2019 in the United States (Siegel et al., 2019). RCC is radiotherapy- and chemotherapy-resistant, and surgery remains first-line therapy (Hsieh et al., 2017; Yin et al., 2019). Despite early surgical treatment, 30% of patients with a localized tumor eventually develop metastases, and 2 years survival rate of patients with metastatic kidney renal clear cell carcinoma (KIRC) is less than 20% (Mickisch, 2002; Janzen et al., 2003). Therefore, identification of underlying molecular mechanisms and metastatic potential of KIRC are essential for improvements in early diagnosis and treatment.

Dysregulation of alternative splicing (AS) is widely considered a new hallmark of cancer and its products are acknowledged as potentially useful biomarkers (Ladomery, 2013). Recent estimates indicate that nine out of every 10 human genes undergo AS in a cell type- or condition-specific manner to create distinct RNA transcripts from the same pre-mRNA molecule (Wang et al., 2008). The key role of AS is further confirmed by the linkage of splicing regulation to numerous human diseases, including neurological disorders and many types of cancer (Scotti and Swanson, 2016). Regulation of AS is a complicated process in which numerous interacting components are at work, including *cis*-acting elements and *trans*-acting factors, complicated by the functional coupling between transcription and splicing (Wang et al., 2015). Corruption of the process may lead to disruption of normal cellular function and eventually disease. Thus, understanding the regulatory patterns that control AS events has the potential not only to give valuable molecular insights but also to provide solutions for various diseases.

AS events are largely controlled by RNA-binding proteins (RBPs) that recognize specific regulatory sequences embedded in the pre-mRNA transcripts (Gerstberger et al., 2014). However, splicing complexes are intricate molecular machines that process tens to hundreds of RNA target genes. At any given time, depending on the context and cellular stimuli, an RBP will affect only a subset of its RNA target genes. This specificity is often provided by a certain factors we named as “modulators,” such as signaling proteins, microRNAs, lncRNAs that control RBPs activity through several different mechanisms, including: expression level (Payne et al., 2018), protein stability and turnover (Garcia-Maurino et al., 2017), nuclear/cytoplasmic localization (Di Liegro et al., 2014), altered protein interactions (Jankowsky and Harris, 2015), and co-transcriptional regulation (Shukla and Oberdoerffer, 2012). Modulators help a cell combine different external signals and make complex downstream decisions. Elucidating their function is necessary for understanding and controlling cell’s response to external stimuli at transcriptional level.

With the increased availability of large data sets derived from high-throughput experiments and computer algorithms, investigating complex transcriptional dysregulation between RBPs and AS events in complex diseases is now possible. Recently, the ENCODE project published eCLIP data sets for 150 RBPs across K562 and HepG2 cell types (Van Nostrand et al., 2016; Yee et al., 2019). Technological advances have made it possible to define the comprehensive target networks of individual RBPs with high accuracy by integrating global splicing profiles upon depletion of each RBP and genome-wide maps of *in vivo*, direct protein-RNA interactions (Zhang et al., 2010; Weyn-Vanhentenryck et al., 2014).

In this study, we established a computational method for dissecting the relationship among RBPs, alternatives splicing events and a kind of proteins that may influenced the splicing regulation effect of RBPs. Our method is unique in its ability to discover how alternative splicing outcomes is changing when modulator expression is different, even though they were regulated by the same RBP. It is the first time in which a triplet describing the relationships among modulators, RBPs and the outcomes of their alternative splicing targets is reported. The triplet contains three objects: a specific RBP, a splicing target regulated by RBP, and a modulator candidate that may change splicing regulation of the RBP. This method was applied to RCC using TCGA-KIRC dataset to identify modulator-dependent RBPs and their targets splicing outcomes in kidney cancer. QKI, as one of the key RBPs in this study, has the greatest number of influenced splicing events. Functional enrichment analysis showed that the inferred QKI modulators were highly associated with regulation progress of some hallmark cancer genes, including ARMH4, LINC01268, PDP2, LAPTM4B, and CD7. The results showed that different expression of modulators was associated with the changing roles of RBPs on regulating their targets alternative splicing outcomes. We expect that such integrated analysis could reveal the roles of RBPs and provide novel insights into understanding alternative splicing mechanisms in kidney cancer.

## Materials and Methods

### Identify Alternative Splicing Events and Gene Expression

Paired-end RNA sequencing data from 480 RCC patients was downloaded from The Cancer Genome Atlas Kidney Renal Clear Cell Carcinoma (TCGA-KIRC). The percentage of inclusion (PSI) of spliced events were derived using Mixture of Isoforms (MISO) (Lee et al., 2013). A PSI value was computed for every identified event in each sample, and the original AS events were further processed to generate high-confidence events by retaining events with a PSI value greater than 0.1 in at least 100 samples from 480 (∼21% samples in total). Then, events that occurred in both the curated and TCGA datasets were retained to form the final set of AS events. In this study, we only focused on skipped exon (SE) alternative splicing events. We defined an altered skipped exon as any exon of a transcript excluding the first and the last exons. Finally, we only kept the events that at least 100 patients’ have PSI value and coefficient of variation (CV) of PSI was larger than 0.1. Gene differential expression analysis was performed using edgeR (Robinson et al., 2010), and CPM was used to estimated gene expression.

### Identify RBPs Targets Using eCLIP Data

We used crosslinking immunoprecipitation (CLIP) data for 150 RBPs profiled in eCLIP peaks (Van Nostrand et al., 2016) downloaded from ENCODE in bed format (Consortium Encode Project, 2012). The peaks in two immortalized human cell types, K562 and HepG2, were filtered by peak enrichment larger than 8 (log_2_FC ≥ 3) and *p* < 10^–5^ as recommended (Van Nostrand et al., 2016). Since the agreement between peaks in two replicates was moderate (the median Jaccard distance 25 and 28% in K562 and HepG2, respectively), we took the union of peaks between the two replicates in both cell lines and then pooled the resulting peaks. We defined an RBP-binding splicing event as the region upstream 300 base pairs of the exon to downstream 300 base pairs of the exon.

### Genomes and Transcript Annotations

February 2009 assembly of the human genome (hg19, GRCh37) was download from Genome Reference Consortium (Church et al., 2011). The respective transcript annotation v19 was download from GENCODE website (Harrow et al., 2012). Transcript annotations were parsed to extract positions of introns and exons.

### Gene Function and Categories Analysis

Functional enrichment analysis was carried out via the hypergeometric test using the clusterProfiler R package. We used Human Gene Ontology annotation provided by Gene Ontology (GO) Consortium (Ashburner et al., 2000; The Gene Ontology Consortium, 2017). GO terms enrichment with adjusted *p* < 0.01 and KEGG pathway enrichment with adjusted *p* < 0.05. The gene types we discussed in this study including immune related genes, which were download from IMMPORT database^1^ and TRRUST v2 database.^2^ The lncRNA gene type annotation was based on biomaRt software suite in R.

### Construction Modulator-RBP-Splicing Triplets in KIRC

The probabilistic model is similar to Li et al. (2017) as follows:

(1) $$Y_{t\,a\,r\,g\,e\,t}=\beta_{0}+\beta_{1}\,X_{r\,b\,p}+\beta_{2}\,X_{m}+\beta_{3}\,X_{r\,b\,p}\,X_{m}+\varepsilon$$

where, the X_rbp_, X_m_, and Y_target_ are the gene expression of RBP and its modulator, and the splicing outcomes of the affected target gene, respectively. X_rbp_ and X_m_ represent the effect of RBP and modulator, respectively, on target by themselves alone (main effects), while β_3_ represents the effect of their interaction. If an RBP and modulator interaction influences target splicing outcomes, we expect β_3_ to be non-zero.

We divide rank-ordered expression values of a gene by tertiles and further discretize the triplets using:

(2) $$x^{\prime }=\left\{ \begin{array}{cc} 1\; & i\,f\,x\,i\,s\,i\,n\,u\,p\,p\,e\,r\,t\,e\,r\,t\,i\,l\,e\\ N\,U\,L\,L\; & i\,f\,x\,i\,s\,i\,n\,m\,i\,d\,d\,l\,e\,t\,e\,r\,t\,i\,l\,e\\ 0\; & i\,f\,x\,i\,s\,i\,n\,l\,o\,w\,e\,r\,t\,e\,r\,t\,i\,l\,e \end{array}\right.$$

Values are ranked and transformed by tertials as follows, and coefficients are estimated from the differences in observed proportions of frequencies:

(1) Splice events are ranked according to PSI.
(2) RBP is ranked by its expression.
(3) Each modulator is ranked by their expression.

After discretization, we only consider the eight bins, where none of the genes has “NULL” value, covering ∼33% of the samples. This simple strategy has been shown to maximize entropy among groups and the selection of significant triplets’ method can be found in Babur et al. (2010).

### Statistical Analysis and Software

The data were analyzed and visualized using R statistics software version 3.4.1 and ggplot2 package. Correlations were assessed using Pearson correlation test. Survival curves were generated by the Kaplan Meier method using the median H-score as the cutoff, and differences were analyzed with the log-rank test.

## Results

### Category of Modulator Action

**FIGURE 1:**
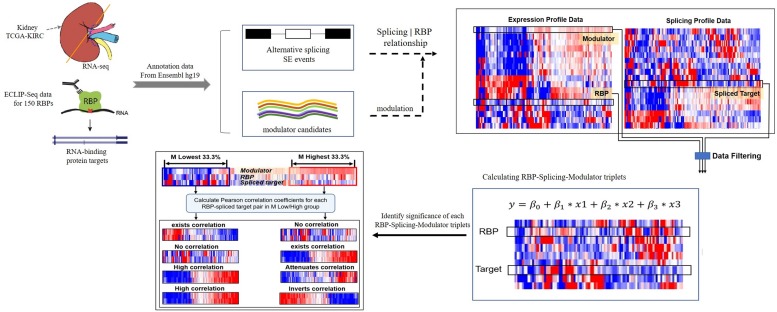
A schematic diagram of workflow. Briefly, the dataset in this study derived from TCGA-KIRC and ENCODE ECLIP-Seq, hg19 was used as reference gene annotation from Ensemble. Each triplet contains three object: RBP, target and a modulator. Gene expression level is the input of RBP and modulator candidates, splicing outcomes (PSI value) is used to estimate the splicing level of target. Data filtering criteria as follows: (1) log2 (CPM) ≥ 1 (2) remove events with “NA” samples > 100 (3) CV(PSI) > 0.1. Then using the linear regression model to predict triplets. Only the triplets with significant β_*3*_ *p*-value will be considered and selected to the following analysis. Finally, for each triplet, we group the samples into “low” and “high” groups based on the expression level of modulator (bottom/top 33% samples) in the specific triplet, and we compare the Pearson correlation coefficient values of RBP expression and target PSI value in two groups, identify the modulator function categories.

We developed a framework to infer the modulators of RBPs whose expression strongly correlates with changes of a RBP’s effect on regulating targets splicing outcomes. Here, the transcriptional activity of a RBP was evaluated by the Pearson correlation between the expression level of RBPs and its target splicing outcomes. A schematic diagram of work-flow is provided in Figure 1.

The proposed method takes five inputs: gene expression profiles, an RBP of interest, a list of modulator candidates, splicing profiles, and RBPs’ binding information. Candidate modulators may include all genes satisfying the criteria. In addition, the expression of the modulator candidates and RBPs were required to be statistically independent. Each possible triplet was then independently tested using the PCCs (Pearson correlation coefficients) estimator, and by comparing Δ*PCCs* we defined the subtype of modulation categories. False positives were controlled using appropriate statistical thresholds. Three possible modes of modulator action were identified, depending on whether RBP-splicing correlation increased or decreased as a function of the modulator abundance.

### Category of Modulator Action

For each triplet, three possible modes of modulator action were identified depending on whether RBP-splicing correlation increased or decreased as a function of the modulator abundance. The three models are “attenuates splicing,” “enhances splicing,” and “‘inverts splicing.” Among them, attenuates/enhances splicing modes including two sub-types: attenuates/enhances exon exclusion and attenuates/enhances exon inclusion; inverts splicing mode means that the mode of modulator switches from exon inclusion to exon exclusion or from exon exclusion to exon inclusion. These cases and details interpretations are listed in Table 1.

**TABLE 1:** Categories of modulator mediated RBP regulations on splicing targets.

Modulation category	PCC_low_	PCC_high_	Δ*PCCs*	Subtype mode
Attenuates splicing	– (EE)	– (EE)	| *PCC*_*low*_| > | *PCC*_*high*_ | or *p-value.high* > 0.05	Attenuates exon exclusion (AEE)
Enhances splicing	– (EE)	– (EE)	| *PCC*_*low*_| < | *PCC*_*high*_| or *p-value.low* > 0.05	Enhances Exon exclusion (EEE)
Inverts splicing	+ (EI)	– (EE)		Exon inclusion to exclusion (ExonIE)
Inverts splicing	– (EE)	+ (EI)		Exon exclusion to inclusion (ExonEI)
Enhances splicing	+ (EI)	++(EI)	| *PCC*_*low*_| < | *PCC*_*high*_| or *p-value. Low* > 0.05	Enhances EI (EEI)
Attenuates splicing	+ +(EI)	+(EI)	| *PCC*_*low*_| > | *PCC*_*high*_| or *p-value.high* > 0.05	Attenuates EI (AEI)

*“+” and “−” signs in the columns indicate positive and negative values of Pearson correlation coefficient. “EE” represents exon exclusion, and “EI” means exon inclusion. The modulation categories of “attenuates splicing.” “enhances splicing” or “inverts splicing” only refer to the roles of RBP on specific alternative spliced exon.*

### Identify Modulators of QKI in Kidney Cancer

We applied the proposed method for identifying modulators for 150 RBPs. 14,707 exons were selected from 42,485 annotated skipped exons that are derived using the gene structures of ENSEMBL database. We identified 1,040,254 significant modulator-mediated triplets from 40,623,520 potential modulator-RBP-splicing interactions at FDR ≤ 0.01 using TCGA-KIRC data. The potential interactions consisted of 137 RBPs, 4,309 splicing events, and 2,905 modulator candidates. Among these RBPs, 13 RBPs were filtered out as the PSI distribution among 480 patients did not meet our criteria (the PSI coefficient of variation should be larger than 0.1). RNA-binding protein Quaking (QKI) had the greatest number (68.9%, 199 out of 289) of modulated spliced exons.

**FIGURE 2:**
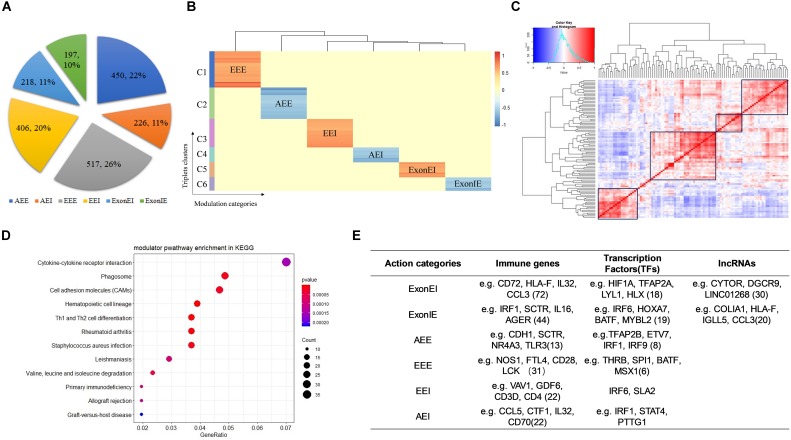
Identify modulators of QKI in KIRC. **(A)** Six mode sub-categories according to the correlation between QKI expression and its target splicing outcomes. The number in the pie chart means the percentage of each sub-categories. AEI indicates attenuates the exon inclusion, AEE indicates attenuates exon exclusion, EEI indicates enhances exon inclusion, EEE indicates enhances exon exclusion, ExonIE indicates reverses exon inclusion to exon exclusion, ExonEI indicates reverse exon exclusion to inclusion. **(B)** Modulators clusters by their regulation patterns. Six clusters were grouped according to modulator sub-categories. **(C)** Correlation heatmap of QKI modulators. The redder the color the higher correlation between two modulators. The values in the matrix were the normalized gene expression of modulators. **(D)** KEGG enrichment analysis of QKI modulators. **(E)** Modulators categories according to gene biotypes and features, including immune genes, transcription factors and lncRNAs.

We identified 2,014 Modulator-QKI-Splicing triplets. The triplets include 1,101 modulators and 187 splicing events corresponding to 130 genes. According to the correlation between QKI expression and its target splicing outcomes, six modulator sub-categories were identified, including 450 triplets in “Attenuates_Exon_Exclusion,” 226 triplets in “Attenuates_Exon_Inclusion,” 517 triplets in “Enhances_Exon_Exclusion,” 406 triplets in “Enhances_Exon_Inclusion,” 218 triplets in “Exon_Exclusion_to_Inclusion,” and 197 triplets in “Exon_Inclusion_to_Exclusion” (Figure 2A).

Furthermore, we observed that most modulators affected multiple splicing targets were multimodal, and the same modulator may play opposite roles on different QKI targets. For example, ARMH4 inverts the splicing activity of QKI on its target CLTC: the inclusion of CLTC’s spliced exon was correlated with increasing expression of QKI when ARMH4 is lowly expressed, while such association was inversed when ARMH4 is highly expressed. However, ARMH4 played enhanced exon inclusion role on QKI-STIM1 pair when its expression level changed from low to high. In another case, while the modulator KRT17 influenced 11 splicing targets of QKI, the role of KRT17 on these splicing outcomes changed among EEE, ExonIE, and ExonEI. The observation indicated that the distinct modulation patterns were triplets dependent rather than depending on specific RBPs or target genes. Our findings support this complexity in modulators typically had many target-specific effects. These findings suggested that more complex models are needed to better elucidate that how splicing is regulated.

Hence, we clustered modulators by their regulation patterns, yielding distinct groups of modulators that mediated splicing dysregulation in specific patterns (Figure 2B). For instance, the modulators in cluster 1–2 tend to reverse the QKI activity on regulating splicing targets’ outcomes, whereas those modulators in cluster 4 and cluster 6 tend to enhance QKI splicing activity. In conclusion, these modulators may work as antagonistic or coactivators to mediate QKI splicing activity.

Among these modulator-mediated triplets, we noticed that many modulators regulate the same QKI splicing targets, this may be because some of the modulators co-express or play similar functions in related pathways. As the result showed in Figure 2C, modulators were grouped into several clusters according to their expression’s correlation. This may be a potential reason why the spliced outcome of same target could be influenced by many modulators. In addition, the pathway enrichment results shown that these modulators were highly enriched in categories which were known to be associated with cancer development and progression, including cytokine-cytokine receptor interaction, Th1 and Th2 cell differentiation, and cell adhesion molecules (Figure 2D).

Furthermore, we classified the modulators of QKI according to gene biotypes and features, including immune related genes, transcription factors and lncRNAs (Figure 2E). The types of categories provide a framework for understanding many types of dysregulation on splicing.

### Functional Analysis of QKI Modulators

To confirm the QKI-splicing-modulator triplet signatures as independent predictors, we selected six inferred modulator-influenced triplets to compare the association among QKI-splicing-modulators. The modulators we focused on were obtained from the analysis result 3.3, including immune genes (CCL3, HLA-F, AGER), transcription factors (ARMH4, STAT4), and lncRNAs (LINC01268).

**FIGURE 3:**
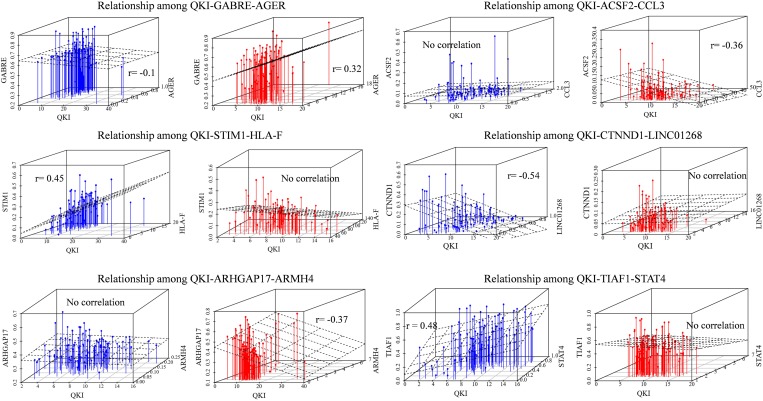
Relationships among QKI-modulator-target. The blue means the samples in modulator expression low group (expression in bottom 33%), the red means the samples in modulator expression high group (expression in top 33%). The correlation value is the Pearson correlation between expression level of QKI and the splicing outcomes of its targets. No correlation means the statistical *p-*value of correlation is not significant (*p*-value cutoff setup as 0.01).

As an example, immune gene AGER as a modulator of QKI, who shown differentially expressed level between cohort and normal samples in KIRC, played inverts exon exclusion role on regulating the splicing outcomes of GABRE. Comparing the two patterns in different groups, when the expression of modulator AGER is low, the PCCs between QKI expression and GABRE splicing level (PSI) is −0.1, while such correlation inverts to 0.32 in another group whose AGER expression high. Similar pattern we found that the correlation between QKI and its splicing target STIM1 was lost from 0.45 to no significant correlation when the modulator immune gene HLA-F expression differentially in two groups. Meanwhile, LINC01268 as modulator played attenuated effect on regulating QKI splicing activity. The correlation between QKI and target CTNND1 was −0.54 in LINC01268 expressed low group, while such correlation gone when LINC01268 expression becomes high (Figure 3).

**FIGURE 4:**
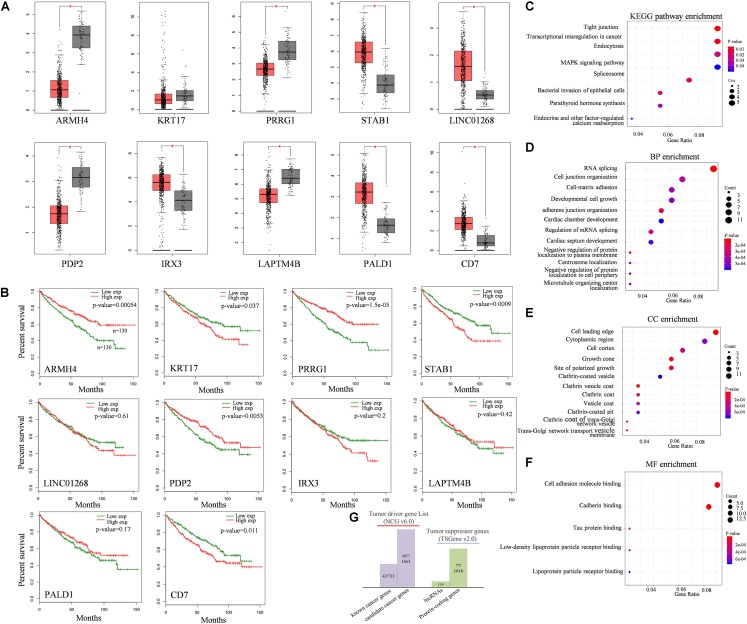
Functional analysis of QKI modulators. **(A)** Expression levels of top10 modulators in KIRC tumor tissues (red boxes) compared with normal tissues (gray boxes**). (B)** survival analysis of top10 modulators. The red line means the modulator expression is high, the green line means the modulator is expression low. Modulator is grouped according to the median value of its expression level. **(C–F)** KEGG pathway and GO enrichment analysis, including biological process (BP), cellular component (CC) and molecular function (MF). **(G)** Cancer-relevant modulators identification according to Network of Cancer genes (NCG) and Tumor suppressor gene (TSGene) database. (*The top10 modulators were selected based on the number of their influenced splicing targets).

To investigate the association between dysregulated target splicing outcomes and kidney cancer, we performed survival analysis and compared the expression level of these modulators in kidney tumor samples and normal samples based on TCGA-KIRC dataset. Results shown that most of the modulators were differentially expressed between tumor and normal samples and overall survival associated. Although KRT17 as one of the modulators we inferred did not show too much differentially expressed, the clinical information obtained from TCGA indicated that gene expression in kidney cancer was significantly associated with overall survival outcomes. The gene expression levels and survival analysis of top 10 modulators who has the most influenced targets of QKI were compared (Figures 4A,B).

KEGG enrichment analysis revealed that these target genes were enriched in categories known to be related to cancer development and progression (Figure 4C), such as tight junction pathway, transcriptional mis-regulation in cancer, endocytosis, and MAPK signaling pathway. The top enriched GO terms of these influenced target genes were associated with transcriptional regulation progress, including RNA splicing, cell growth and protein binding (Figures 4D–F). The results were reasonable as QKI regulated its target mainly on splicing level, once the expression of QKI was perturbed by the modulators, the roles of QKI on its targets, including binding, splicing, cellular development and transcriptional regulation would be influenced consequently.

In addition, cancer-relevant modulators were identified though tumor associated gene list from the Network of Cancer Genes (NCG, v6.0) database (Repana et al., 2019) and Tumor suppressor gene database (TSGene v2.0) (Zhao et al., 2016), separately (Figure 4G). The 2,372 tumor diver genes obtained from NCG including 711 known cancer genes and 1,661 candidate cancer genes. Among them, 149 genes overlapped with tumor diver genes, almost reaching 13% (149/1,179) of total numbers of modulators we inferred in this study. Meanwhile, approximately 7% (77/1,179) modulators were tumor suppressor genes, and the gene type was protein coding gene.

### Analysis the Splicing Outcomes of CTNND1 Influenced by Modulators in Kidney Cancer

**FIGURE 5:**
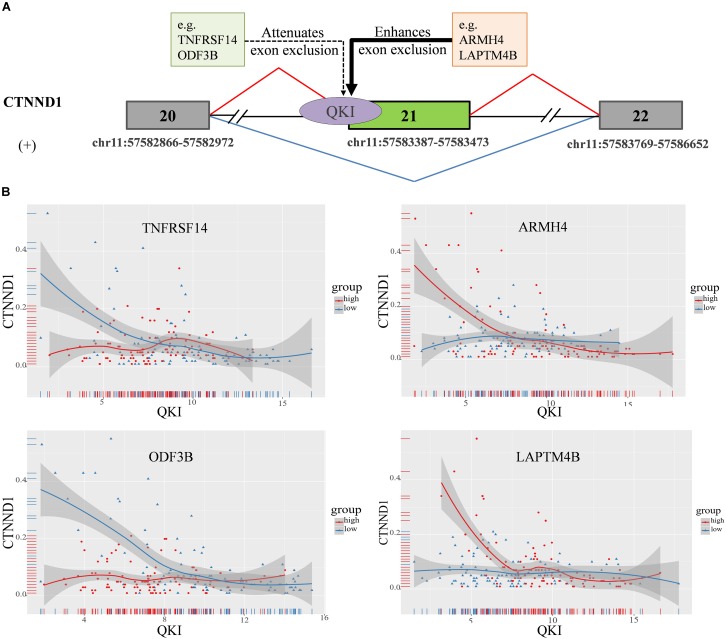
CTNND1 splicing outcomes influenced by modulator expression in KIRC. **(A)** Examples of modulators in each mode sub-categories, including exon exclusion to inclusion (ExonEI), exon inclusion to exclusion (ExonIE), enhances exon exclusion (EEE) and attenuates exon exclusion (AEE). **(B)** Four modulators influence splicing outcomes of CTNND1, including TNFRSF14, ARMH4, LAPTM4B, and ODF3B. The red means the samples in modulator expression high group (top 33%), the blue means the samples in modulator expression low group (bottom 33%). The x-axis is the expression level of QKI, y-axis is splicing outcome (PSI value) of CTNND1.

In this study, we found that the spliced outcome of the 20th exon of CTNND1 has the most inferred modulators, including 30 lncRNAs and 80 protein coding RNAs. The corresponding AS event is “chr11:57582866: 57582972: + @ chr11: 57583387: 57583473: + @ chr11:57583769: 57586652:+.” Previously study reported that CTNND1 encodes a member of the Armadillo protein family, which function in adhesion between cells and signal transduction (Zhu et al., 2012), multiple CTNND1 isoforms are expressed in cells via alternative splicing, only full-length CTNND1 promotes invasiveness (Yanagisawa et al., 2008). Two modulation categories were identified including 107 attenuates exon exclusion (AEE), 7 enhances exon exclusion (EEE). The alternative spliced exon of CTNND1 and some of it’s in each category are shown in Figure 5A.

For example, TNFRSF14 as one of modulators of QKI attenuates the splicing regulation on the 20th exon exclusion of CTNND1. We found that, in TNFRSF14 expression low group, the correlation between QKI expression and CTNND1 PSI is −0.38 (*p* = 1.7e−05), while such correlation is lost when in TNFRSF14 expression high group (correlation = 0.001, *p* = 0.98). This indicated that high expression of modulator TNFRSF14 may play negative effect on changing the splicing activity of QKI. In addition, we found that LAPTM4B, as another modulator of QKI, played enhanced exon exclusion role on regulating the 20th exon splicing outcome when it expression is high (Figure 5B). Thus, the results showed that differentially expressed modulators indeed changed the role of QKI on regulating CTNND1’s splicing outcomes, and we believed that this kind of regulation may provide important insights for study dysregulation of splicing outcomes associated with various diseases.

## Discussion

Alternative splicing alterations may confer a selective advantage to the tumor, such as angiogenesis (Amin et al., 2011), proliferation (Bechara et al., 2013), cell invasion (Venables et al., 2013), and avoidance of apoptosis (Izquierdo et al., 2005). Some splicing mRNA isoforms could change the reading frame, resulting in the generation of different protein isoforms with diverse functions and/or localizations (Sutandy et al., 2018). One of the traditional methods to estimate the functions of mRNAs or protein is comparing the difference of gene expression level (Kim et al., 2014; Lorthongpanich et al., 2019; Xu et al., 2019).

However, not all detected alternative splicing events might necessarily result in mRNAs or proteins expression level changing. In addition, global description of alternative splicing networks and demonstration of their functional consequences have now emerged as one of the biggest challenges of the field (Baralle and Giudice, 2017). By integrating gene expression profile with splicing outcomes of alternative splicing events may be one of the possible ways to study the functional consequences for most of the identified splicing events.

In this study, we established a computational method for identification the modulators whose expression is associated with changing the targets splicing outcomes of RBPs in KIRC. Previously, several computational methods have been developed to identify modulators associated with transcription factors (TFs) regulation activity on expression level (Wang et al., 2009; Babur et al., 2010; Li et al., 2016, 2017), these studies discussed the transcriptional activities of TFs can be influenced by the expression level of modulators. Our method is unique in its ability to discover how alternative splicing outcomes is changing when modulator expression is different, even though they were regulated by the same RBP. And the method aimed at dissecting the effects of disruption in RBPs and hopefully it could provide insight into studying alternative splicing networks during development, cell differentiation, and in disease.

During tissue development and cell differentiation specific RBPs are finely regulated at their expression levels, localization, their own splicing, mRNA stability, and translation efficiency (Baralle and Giudice, 2017). RBPs bind to *cis-*elements promoting or inhibiting splice site recognition, hence RBP expression coordinates alternative splicing networks during development. We focused on 150 RBPs in this study, and only 137 RBPs remained in the final analysis due to there was no splicing targets of them within our criteria. Three possible modes of modulator actions were defined in this study, depending on the correlation changes between RBP and its target splicing outcomes when modulator expression is different. Among these RBPs, we found that QKI had the greatest number of influenced spliced exons (68.9%, 199 out of 289), and 2,014 Modulator-QKI-Splicing triplets were finally identified focused on QKI. Results showed that most modulators affected multiple splicing targets were multimodal, and the same modulator may play opposite roles on different QKI targets.

For example, high expression level of modulator ADAM8 enhanced QKI role on regulating ACSF2 exon exclusion, while it enhanced target FMNL2 exon inclusion in regulating splicing outcomes. Another example, low expression level of AJM1 modulated QKI attenuated exon exclusion on regulating CTNND1 splicing outcomes, while such modulation role changed into enhanced exon inclusion when the target became ATF2. The detailed information about the modulators roles in the triples could be found in Supplementary Table S1.

Pathway enrichment results showed that all these influenced splicing target events of QKI were enrolled in cancer development and progression related pathways, including tight junction pathway, transcriptional mis-regulation in cancer, endocytosis, and MAPK signaling pathways. This evidence indicated that these alternative spliced events played crucial roles in kidney cancer, and changes the splicing outcomes of them may result in dysregulation in alternative splicing networks.

Among all these influenced splicing events, CTNND1 attracted more attention as the splicing outcomes of the 20th exon has the most inferred modulators. Previously study reported that CTNND1 was a tumor-driver gene, whose alternative splicing was related to cell invasion and metastasis (Yanagisawa et al., 2008). In addition, CTNND1 (p120) consists of central ARM domain flanked by the N-terminal regulatory (NTR) and C-terminal tail region (CTR) (Ishiyama et al., 2010), and the 20th exon of CTNND1 is in CTR region. Thus, different splicing outcomes of CTNND1 may influence the domain function, resulting in the generation of different protein isoforms with diverse functions.

We identified 114 inferred modulators of QKI-CTNND1 pair, including 30 lncRNAs and 80 protein-coding RNAs. Han et al. (2016) reported that MALAT1 may play as a tumor-suppressor gene in gliomas, and high MALAT1 expression linked to cell proliferation and metastasis. In our results, we noticed that high expression of modulator MALAT1 tended to attenuate QKI regulation role on splicing the 20th exon exclusion in CTNND1. The correlation between QKI expression and CTNND1 PSI was −0.51 (*p* = 4.3e−09) in MALAT1 expression low group, and such correlation changed into −0.20 (*p* = 0.02) in MALAT1 expression high group. LINC00174 as another inferred modulator had been reported that it exerted a tumorigenesis role in glioma. LINC00174 knockdown inhibited cell proliferation, migration, invasion and glycolysis (Shi et al., 2019). We found that, when LINC00174 expression is low, the correlation between QKI expression and splicing outcomes of CTNND1 is −0.37 (*p* = 2.6e−05), while such correlation was lost (*r* = −0.07, *p* = 0.44) when LINC00174 expression became high. These evidences showed that regulation of alternative splicing outcomes is a complex progress, it different splicing consequence not only associated with RBP but also associated with other proteins expression.

Although the established model in this study and the corresponding results appear helpful for understanding the alternative splicing regulation, there are some limitations. First, many proteins tended to show similar expression pattern, certain RBP-target pairs may have more than two inferred modulators, these results may contain certain false positive modulators. In addition, the function and mechanism of how modulator changes the RBPs regulation on their target splicing outcomes need to be further studied by experiments, for example, modulator co-expressed or physically interacted with RBPs, and this is a long way for us to go. Finally, we expect that our study could provide novel insights for understanding the dysregulation of alternative splicing in cancer.

## Data Availability Statement

Paired-end RNA sequencing data from 480 RCC patients was downloaded from the Cancer Genome Atlas Kidney Renal Clear Cell Carcinoma (TCGA-KIRC).

## Author Contributions

YL and YW designed the study. YW and SC wrote the code, analyzed data, and wrote the manuscript. XR calculated the alternative splicing events in KIRC. All authors read and approved the final manuscript.

## Conflict of Interest

The authors declare that the research was conducted in the absence of any commercial or financial relationships that could be construed as a potential conflict of interest.

## Footnotes

1 https://www.immport.org

2 https://www.grnpedia.org

## Supplementary Material

TABLE S1 Inferred QKI modulators and triplets’ information.
